# Development of Two Types of Skin Cancer in a Patient with Systemic Sclerosis: a Case Report and Overview of the Literature

**DOI:** 10.1155/2021/6628671

**Published:** 2021-02-26

**Authors:** Firdevs Ulutaş, Erdem Çomut, Veli Çobankara

**Affiliations:** ^1^Division of Rheumatology, Department of Internal Medicine, Pamukkale University Faculty of Medicine, Denizli, Turkey; ^2^Department of Pathology, Pamukkale University Faculty of Medicine, Denizli, Turkey

## Abstract

Systemic sclerosis (SSc) is an uncommon rheumatic disease in which the underlying main histopathologic feature is a thickening of the skin due to excessive accumulation of collagen in the extracellular tissue. Fibrogenesis, chronic inflammation, and ulceration may eventually promote skin neoplasms. Although nonmelanoma skin cancer (NMSC) is the most frequent type, there have been restricted case reports and case series with skin cancers in SSc patients in the literature. Herein, we describe a 78-year-old woman diagnosed with diffuse cutaneous systemic sclerosis thirteen years ago and associated nonspecific interstitial pneumonia that was successfully treated with high cumulative doses of cyclophosphamide. She developed basal cell carcinoma and squamous cell carcinoma of the skin in the follow-up. She is still on rituximab treatment with stable interstitial lung disease as indicated by pulmonary function tests and high-resolution chest computed tomography. To our knowledge and a literature search, this is the first reported patient with SSc with two types of skin cancer. In this review, we also aimed to emphasize the relationship between SSc and skin cancer, and possible risk factors for SSc-related skin cancer.

## 1. Introduction

Systemic sclerosis (SSc), also called scleroderma, is an uncommon rheumatic disease. It is a slowly progressive disease that is symbolized by vasculopathy, fibrosis of the skin and visceral organs, immune rearrangement, and B-cell activation with characteristic autoantibodies [1]. Although evidence for high risk of having malignancy is suggestive, the types of malignancies and the importance of the total risk are quite variable in each patient with SSc [2]. The lung and skin are the frequently seen specific tumor sites in patients with SSc, which are commonly affected by both fibrosis and underlying immune dysfunction [3]. Interstitial lung disease (ILD) is also a serious complication of SSc, which leads to significant morbidity and lung cancer [4]. Autoantibodies to topoisomerase I (also referred to as anti-Scl70), smoking, and some immunosuppressive drugs may also lead to a higher frequency of lung cancer among SSc patients [5]. On the other hand, skin cancers are also seen in SSc patients. Although nonmelanoma skin cancer (NMSC) is the most frequent subtype, there have been restricted case reports and case series with skin cancers in SSc patients. Although underlying pathogenetic mechanisms are not yet clear, a shred of evidence is present related to few immunosuppressive drugs. Cyclophosphamide (CyP) is one of them and nonselectively inhibits the whole immune system, and malignancy may develop by suppressing immune surveillance [6]. Today, the use of CyP for the treatment of ILD in connective tissue disorders has growing evidence for optimization of lung functions [7]. Immunosuppression with methotrexate use alone and/or combined with antitumor necrosis factor agents (anti-TNF) is also related to high risk of second nonmelanoma skin cancers (NMSC) in rheumatoid arthritis (RA) patients [8].

Herein, we report an older SSc patient who was treated with highly toxic cumulative doses of CyP due to progressive ILD and who subsequently developed two types of skin cancer simultaneously. Managing treatment of individuals with SSc-ILD is difficult due to balancing the need for therapy in severely progressive patients against the potential for adverse effects. Clinicians should identify patients who will develop the progressive disease with the lowest level of unexpected side effects before planning treatment. In addition to treatment modalities, underlying inflammatory diseases, prolonged life span and family histories, occupations, and sun exposure of patients should be examined. Written informed consent was obtained from our patient for publication.

## 2. Methods

A literature search was performed in PubMed using these terms: “scleroderma,” “systemic sclerosis,” “skin cancer,” “squamous cell carcinoma,” and “basal cell carcinoma.” An English language filter was activated. All case reports published before November 2020 were examined.

## 3. Case Report

**Figure 1:**
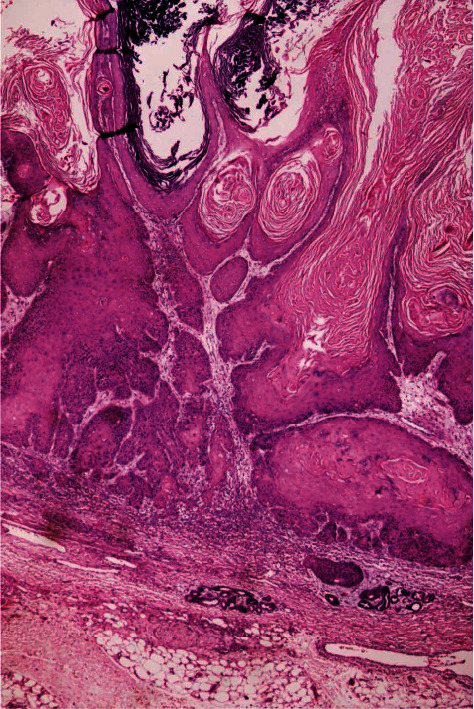
Squamous cell carcinoma. Blunt-type intradermal invasion of well-differentiated tumor nests (H&E, ×40).

**Figure 2:**
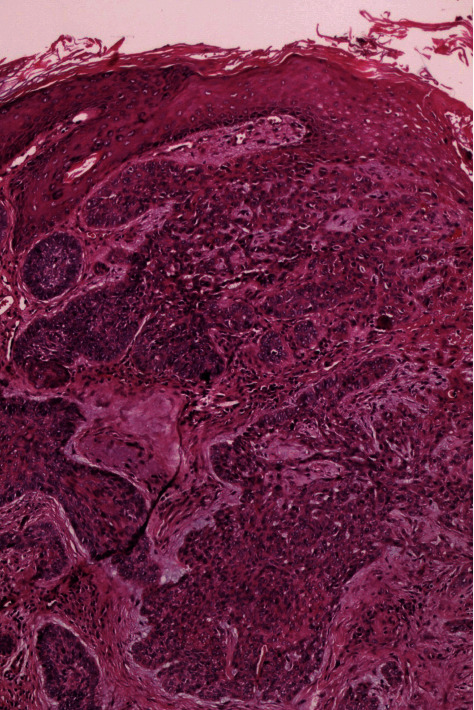
Basal cell carcinoma. Nests of basaloid cells project from the overlying epidermis (H&E, ×40).

A 78-year-old woman diagnosed with diffuse cutaneous systemic sclerosis (dcSSc) thirteen years ago is on regular follow-up in the tertiary health center, in Denizli. She is of Turkish origin and works as a farmer for a long time. She reports no use of tobacco and has no known allergic diseases. Her past medical history included idiopathic venous thromboembolism in 2016 without an identifiable cause. She was diagnosed with dcSSc based on the presence of Raynaud's phenomenon, digital pitting scars, sclerodactyly, and diffuse skin sclerosis extending proximal to the metacarpophalangeal joints on both hands. An indirect immunofluorescence test for antinuclear antibodies (ANA) was positive (titer ≥ 1 : 1280), and anti-Scl-70 was also positive. In the first year of diagnosis, she worsened and complained of dyspnea occurring with minimal exertion, along with chronic, persistent cough. Transthoracic echocardiogram revealed no findings suggestive of pulmonary arterial hypertension. After spirometry tests (forced vital capacity of 1.79 L (40% of the predicted)), high-resolution chest computed tomography (nonspecific interstitial pneumonia (NSIP)), and a 6-minute walk test (developing desaturation at 424 m), she was accepted as having SSc-related NSIP. CyP was administered at a dose of 1000 mg/m2 of body surface area per month for six months in an outpatient clinic, followed by 500 mg/m2 of body surface area every two months for one year. After two years of clinical stability, the same treatment regimen was repeated due to signs of lung exacerbation (total cumulative dose has exceeded 15 g). After three years of completed treatment, a 1-2 cm nonhealing ulcer on her right forearm and a pigmented mass at the tip of the nose appeared and were excised surgically. Histopathological examinations revealed squamous cell carcinoma (SCC) and basal cell carcinoma (BCC), respectively (Figures 1 and 2). Surgical borders were intact without tumor cells. Additional scanning methods showed no local or distant metastases. She is still on rituximab treatment with stable interstitial lung disease as indicated by pulmonary function tests and high-resolution chest computed tomography.

## 4. Discussion

Skin cancers, covering SCC, BCC, and malignant melanoma were found as SSc-related tumors in a literature search. Although a malignant turn of localized scleroderma is rarely seen, Durcanska et al. concluded that a young 26-year-old woman who was diagnosed with localized scleroderma (morphea) ultimately developed SCC camouflaged by osteomyelitis on the lower extremities after a long course of the disease [9]. An 18-year-old woman with progressive SSc developed SCC of the skin. Despite resection, the tumor recurred and was resistant to local radiotherapy [10]. Song et al. reported a 46-year-old man with systemic sclerosis and BCC on his face that was successfully removed by surgical excision [11]. Sargın et al. reported variable cancers in 7 cases among 153 systemic sclerosis patients. Two of all the cancers were malignant melanoma in the eyes and skin, in Aydın, neighboring to Denizli [12]. Koksal et al. investigated the distribution of cancer cases in Denizli between 2000 and 2004. 10.9% of 2185 cancer cases were skin cancers. They emphasized a significant increase in skin cancers over the years [13]. Ceylan et al. investigated features of NMSCs in Izmir where high ultraviolet light exposure was present such as Denizli. Tumors were commonly seen in elderly men and were also related to sun exposure and older age. Among 3,186 NMSC lesions, 71 patients had both BCC and SCC, and BCC was the most common type among the whole group, and mostly located on the face [14]. Nose and lip were the most common locations on the face with high recurrence rates for NMSC [15]. Our patient also had SCC and BCC on the tip of her nose and on her right forearm which may be related to sun exposure.

The main histopathologic feature of scleroderma is a thickening of the skin due to excessive accumulation of collagen in the extracellular tissue. Fibrogenesis, chronic inflammation, and ulceration may eventually promote skin neoplasms [16]. SCC has been the most frequently reported skin cancer in association with chronic tissue inflammation [17]. Also, Magro et al. stated that not only malignancies but also atypical lymphoid aggregates are seen in cutaneous specimens of connective tissue disease (CTD) related to B-cell or T-cell phenotypes [18]. Numerous theories and pathophysiological mechanisms have been emphasized to explain this clinical association. One of the highlights is a significantly reduced percentage of CD4(+) Foxp3(+) T(reg) in the skin of patients with SSc or limited scleroderma [19]. Dysregulation of the endocannabinoid system (ECS) is also related to skin cancer in scleroderma [20]. Recent extensive research points to the renin-angiotensin-aldosterone system- (RAAS-) modulating drugs in the impaired regulatory function of local RAAS to be related to cancer development and scleroderma-like skin changes [21]. Recent literature revealed that the prevalence of all epithelial skin neoplasms, involving melanoma, SCC, and BCC, was significantly higher in patients with morphea compared with the healthy subjects [22]. Besides, a study in which malignancies were analyzed in SSc patients and were compared with the general population revealed 11 new cases of variable malignancies in 10 SSc patients (4.6%) more commonly seen than the general population. Only one of them had skin cancer [23]. Although there is no comprehensive analysis of the prevalence of skin cancers and other malignancies among SSc in our health center, we can report that she is the first case with two concurrent types of skin cancer.

SSc-specific autoantibodies may identify patients at high risk and play a role in the prognosis and triaging of patients who may require further cancer screening [24]. However, the best-known autoantibodies such as antitopoisomerase I and anticentromere are inconsistent in predicting risk for developing malignancy. In a comprehensive review, increased age, diffuse SSc, and female gender were well-known risk factors for the development of malignancy in patients with SSc [25]. In another large cohort including 2177 patients with SSc, the presence of anti-RNA polymerase III (anti-RNAPIII) was the most associated antibody for the development of malignancies [26]. Female gender, older age, and having antitopoisomerase I antibody were personal, unchangeable risk factors in our patient.

Also, immunosuppressants used in major organ involvements of scleroderma may encourage tumor formation. CyP is a very efficient immunosuppressant drug in autoimmune and inflammatory illnesses with possible major side effects [27]. Hematological adverse events, bone marrow suppression, hematuria, bladder cancer, and gonadal damage have been demonstrated and are the most feared side effects [28]. Increased risk of hematological and solid organ malignancies in patients treated with CyP suggests carcinogenic activity and should be kept in mind [29]. However, in addition to immunosuppressive drugs, some immunosuppressive conditions such as HIV, chronic lymphocytic leukemia, and/or transplant recipients have also a higher risk for developing any skin cancer than the general population [30].

Other important points are sun exposure and advanced age in the pathogenesis of NMSC. The most common cancer in the world, NMSC, was developed more commonly in outdoor workers including different job groups than indoor workers due to sun exposure in a recently published paper [31]. Although it is difficult to clearly distinguish the causative etiopathogenic mechanism, our patient had apparent multiple risk factors for developing skin cancer, including high sun exposure due to her occupation, exposure to high cumulative toxic doses of CyP, long-term immunosuppression with CyP and rituximab, the underlying chronic inflammatory disease, systemic sclerosis, and the advanced age. We think the unfortunate outcome in this patient is the cumulative result of all these risk factors.

## 5. Conclusion

We reported a rare case of BCC and SCC in a SSc patient along with a review of the literature. To our knowledge and the literature search, this is the first reported SSc patient with two types of skin cancer. With this review, we aimed to improve our knowledge of SSc-related skin cancers. Clinicians should always keep in mind the patient's risk of developing toxicity based on a risk-benefit analysis. These patients are candidates for cancer development due to underlying immune dysregulation and immunosuppressive drugs, in addition to traditional risk factors.
